# Underestimation of extremes in sea level surge reconstruction

**DOI:** 10.1038/s41598-024-65718-6

**Published:** 2024-06-27

**Authors:** Ludovic Harter, Lucia Pineau-Guillou, Bertrand Chapron

**Affiliations:** 1 IFREMER, Laboratoire d’Océanographie Physique et Spatiale, UMR 6523 (IFREMER, CNRS, IRD, UBO), IUEM, Brest, France; 2 IRD, Sorbonne University, Institute of Ecology and Environmental Sciences of Paris (iEES), UMR 7618 (Sorbonne Université, UPCité, UPEC, CNRS, INRAE, IRD), Paris, France

**Keywords:** Physical oceanography, Physical oceanography

## Abstract

Statistical models are an alternative to numerical models for reconstructing storm surges at a low computational cost. These models directly link surges to metocean variables, i.e., predictors such as atmospheric pressure, wind and waves. Such reconstructions usually underestimate extreme surges. Here, we explore how to reduce biases on extremes using two methods—multiple linear regressions and neural networks—for surge reconstructions. Models with different configurations are tested at 14 long-term tide gauges in the North-East Atlantic. We found that (1) using the wind stress rather than the wind speed as predictor reduces the bias on extremes. (2) Adding the significant wave height as a predictor can reduce biases on extremes at a few locations tested. (3) Building on these statistical models, we show that atmospheric reanalyses likely underestimate extremes over the 19th century. Finally, it is demonstrated that neural networks can effectively predict extreme surges without wind information, but considering the atmospheric pressure input extracted over a sufficiently large area around a given station. This last point may offer new insights into air-sea interaction studies and wind stress parametrization.

## Introduction

In the North-East Atlantic, coastlines are frequently impacted by extreme sea levels. The impact of flooding events may be dramatic in terms of the economy, environment and loss of human life. Around the world, one billion people live on land less than 10 m above current high tide lines, including 230 million people living on land less than 1 m^1^. In an evolving warming climate, better predicting extreme sea levels is essential for estimating the future risks of flooding and protecting vulnerable coastal communities.

Storm surges are mainly associated with low-pressure systems that bring strong winds and waves. Storm surges show considerable interannual to multidecadal variability^2,3^. Consequently, long records (i.e., centennial) are needed to infer long-term trends possibly linked with global warming rather than short-term trends reflecting multidecadal variability. Long records may come from (1) observations, such as those from tide gauges^2,4,5^ and altimeters^6^, or (2) numerical hindcasts^7,8^. However, (1) observations partly suffer from inadequate spatial or temporal resolution, the presence of gaps, and limited spatial coverage, and (2) numerical hindcasts can be time-consuming to implement and computationally expensive, as high resolution is needed in coastal areas. For these reasons, statistical models based on metocean data (observations or hindcasts) have been developed to reconstruct long storm surge records with minimal computational cost^6,9–11^.

In the literature, statistical reconstructions are mostly based on multiple linear regression or machine learning approaches. Multiple linear regressions were used long before the numerical era to infer storm surges from meteorological conditions^12,13^ and are still largely used for storm surge reconstruction^2,9–11^. Meanwhile, machine learning methods, and more specifically deep learning approaches, have emerged, demonstrating great potential for statistically modeling extreme sea levels. Tadesse et al.^11^ used a technique based on random forest, a supervised machine learning algorithm, to model storm surges globally. Bruneau et al.^14^ applied neural networks techniques to estimate global sea level extremes. Tiggeloven et al.^15^ used four types of neural networks, and compared their performances to model storm surges in coastal areas. Ramos-Valle et al.^16^ and Lockwood et al.^17^ implemented artificial neural networks to predict hurricane storm surges. Some of these studies compared two approaches: multiple linear regressions versus machine learning. Tadesse et al.^11^ implemented multiple linear regressions and a random forest approach globally, and selected the best-performing method for each station. The linear approach was preferred at only 12% of the stations. Bruneau et al.^14^ compared neural networks versus multiple linear regressions and concluded that neural networks performed best. However, attention must be devoted to the choice of the predictors (i.e., input metocean data) to take advantage of the potential of machine learning methods.

Key predictors of storm surge statistical models are generally the atmospheric pressure and the wind. Both can be introduced in different ways (e.g., raw data, gradients, squares), depending on the study. The atmospheric pressure effect is classically introduced through sea level pressure data^10,11^. In some studies, its gradient has also been considered^9,14^, and more rarely, its squared gradient^9^. The wind effect is generally introduced through the 10 m wind speed (both components) or through the wind stress, that is, at first order, proportional to the square of the wind speed. Surprisingly, there is no consensus on which is the best predictor between wind speed and/or wind stress. Arguments in favor of wind stress highlight its physical mechanism to drive an enhanced Ekman setup over coastal region located to the right-side (North-Hemisphere) of the storm trajectory. Almost 60 years ago, Sutcliffe and Lennon^12^ correlated the storm surges along the west coast of the British Isles with the “tractive force of the wind” (i.e., the wind stress) rather than the wind. At the beginning of the 1980s, Amin^13^ analyzed the linear relationship between storm surges and wind at tide gauges located on the west coast of Great Britain. He concluded that, depending on the location, surges are either proportional to wind or the square of wind velocity. Currently, both predictors are used; some studies rely on wind speed as a predictor^9,10,14,15,17^, whereas others prefer wind stress^2,11,18^. In addition to these classical predictors (atmospheric pressure and wind), other predictors may be considered, such as waves (significant wave height, peak period), precipitation or sea surface temperature, to try to model omitted processes such as wave setup (additional surge due to wave dissipation in nearshore areas) or river-flow induced surge. For example, Tadesse et al.^11^ considered sea surface temperature and precipitation as additional predictors, whereas Bruneau et al.^14^ considered precipitation and waves as supplementary predictors. However, the contributions of these additional variables (e.g., waves, precipitation, sea surface temperature) on the performance of the model have not been investigated, to the best of our knowledge.

Finally, regardless of the method, storm surge reconstructions generally underestimate the most extreme events^10,14,16,19^. The reasons may be model inaccuracies but also inappropriate formulations of the drivers, omitted storm surge drivers and underestimation of atmospheric input data (for example, the wind). In addition, extremes are by definition rare in the dataset, which makes training more difficult for statistical models. To generate coherent storm surge datasets, this bias is often removed. Cid et al.^9^ applied a quantile-mapping bias correction, whereas Ji et al.^10^ implemented a Geographical Differential Analysis (GDA) calibration.

In this study, the objective is to further investigate the underestimation of extremes by exploiting two methods for storm surge reconstruction in the North-East Atlantic: multiple linear regressions and Neural Networks (NNs). The paper addresses the following questions: (1) which predictor among wind stress and wind speed best reduces the bias on extremes? (2) Does the addition of the waves as a predictor reduce the bias on extremes? (3) Do historical atmospheric reanalyses accurately represent extremes?

## Data

### Sea level data

**Figure 1:**
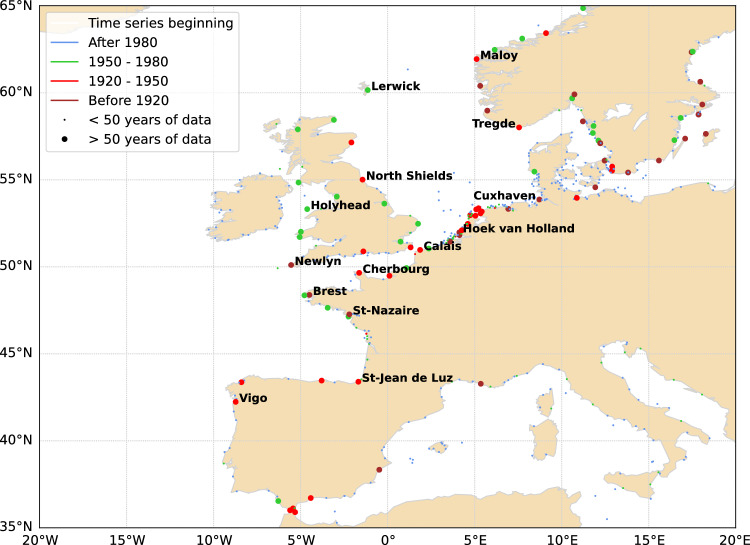
Location of GESLA-3 stations along the North-East Atlantic coastlines. The stations named are those used in this study. Map generated using Python Matplotlib 3.5.3 (https://matplotlib.org/).

We used water level data at tide gauges from the GESLA-3 (Global Extreme Sea Level Analysis Version 3) database^20–22^. This dataset provides high-frequency water level data (i.e., hourly or more frequent) at 5,119 stations worldwide. Some stations recorded long time series, i.e., longer than 100 years. For instance, the tide gauge of Brest (France) is the second oldest in the world and covers 176 years of hourly data (since 1846). Here, we selected 14 stations that are spatially evenly distributed in the North-East Atlantic and covered more than 50 years (Fig. 1). Their characteristics (time span, number of years of data) are summarized in Supplementary Table 1.

The surges were computed hourly and calculated by removing the predicted tide and the mean sea level contribution from the total sea level. The tide was computed for the last 20 years of each record using the Tidal Toolbox^23^. We carefully removed the harmonic constituents Sa (annual) and Ssa (semi-annual) for the tide prediction, as these two seasonal components reflect the radiational tide (atmospheric-driven variations) rather than the gravitational tide (due to gravitational forces of the Moon and Sun). Note that Sa is purely radiational (i.e., not present in the tidal potential), whereas Ssa is mainly radiational (with a small contribution in the tidal potential^24,25^). Removing Sa and Ssa from the tide ensures that the seasonal cycle is fully retained in storm surges. The mean sea level contribution was removed by taking off the annual mean sea level, computed as the average of hourly sea level data over a year. This is essential to ensure that the mean sea level rise is not included in the residual surge signal^5,26^. Hourly surge time series at the 14 selected stations are then obtained.

### Atmospheric data

We used atmospheric data (sea level pressure and 10-meter wind components) from two reanalyses: ERA5 from ECMWF^27,28^ and Twentieth Century Reanalysis version 3 (20CRv3) from NOAA^29,30^. ERA5 provides hourly global atmospheric variables on a 0.25$^{\circ }$ $\times$0.25$^{\circ }$ grid from 1979 to the present. Note that ERA5 starts in 1940, but only the period 1979 onwards was publicly available at the time of the analysis. The 20CRv3 reanalysis provides 3-h estimates of global atmospheric variables across a 75 km grid from 1836 to 2015.

We extracted the atmospheric variables locally, i.e., at the nearest grid point to the tide gauge, or regionally, i.e., on a box around the tide gauge (1$^{\circ }$ $\times$1$^{\circ }$, 3$^{\circ }$ $\times$3$^{\circ }$ and 6$^{\circ }$ $\times$6$^{\circ }$). When the data were extracted locally, we carefully selected the grid points at sea (not on land) because surface wind speed decreases significantly on land.

### Wave data

We used wave data (significant wave height) from the Ifremer ResourceCode dataset^31,32^. ResourceCode provides sea-state hindcasts from 1994 to 2020 on an high resolution unstructured grid over the European Shelf (with a resolution of approximately 500 m along the coasts). The hindcast was generated with WAVEWATCH-III model^33^, which was forced with ERA5 winds. Note that the wave model bathymetry needs to be accurate, to properly account for refraction and dissipation phenomena. Here, the bathymetry of WAVEWATCH-III has a very high spatial resolution (around 100 m). It combines the HOMONIM dataset^34^ which covers the Channel and the Bay of Biscay with a resolution of approximately 100 m, and the EMODnet dataset^35^ which covers the Europe’s seas with a resolution of around 200 m. This high resolution bathymetry enables the wave model to perform very well (RMSE of around 0.25 m between model and observations in nearshore areas).

Wave data were always extracted locally. For each tide gauge, we chose the point of extraction as close as possible to the station, but preferentially located in the surf zone where the waves are breaking and could contribute to the wave setup. Several locations for the point of extraction have been tested, without leading to different results. Note that the significant wave height is used in this study as a first order proxy for wave setup. Other variables, possibly controlling the near-shore wave dissipation such as the peak period or the beach slope, may also play a role in wave setup but are not considered here.

## Results

### Underestimation of extremes

**Figure 2:**
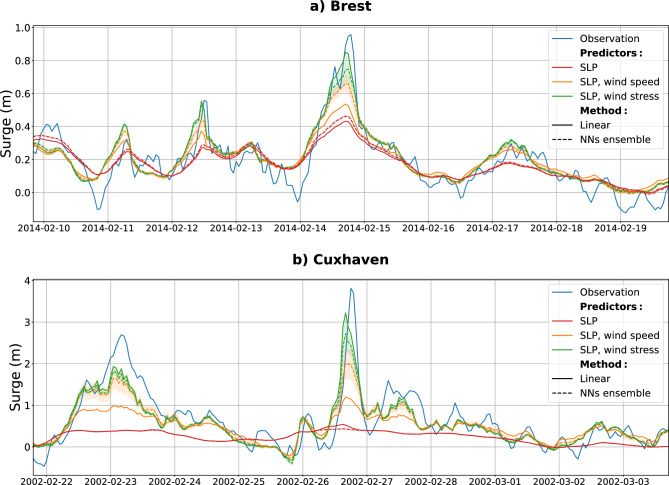
Reconstruction of the strongest storm surge events since 1994 at (**a**) Brest (storm Ulla, 14 Feb. 2014) and (**b**) Cuxhaven (storm Anna, 26 Feb. 2002). Two different methods are employed: a multiple linear regression (solid line) and Neural Networks (dashed line). Various local predictors are used: SLP only (red), SLP combined with wind speed (orange) and SLP combined with wind stress (green). For the NNs, the dashed line represents the ensemble mean, the shaded areas depict the standard deviation of the ensemble (comprising 20 members). Observations are represented in blue.

We reconstructed hourly surges over the period 1994–2020 at the 14 tide gauges with two statistical methods (multiple linear regressions and neural networks, more details in the Methods section) and with various predictors: SLP (Sea Level Pressure) only, SLP and wind speed, SLP and wind stress. All these predictors were extracted locally from the ERA5 renalysis dataset. To illustrate the capacity of the models to reproduce storm surges, the strongest storm surge event since 1994 occurring at Brest (storm Ulla on 14 February 2014) and Cuxhaven (storm Anna on 26 February 2002) are presented in Fig. 2.

**Figure 3:**
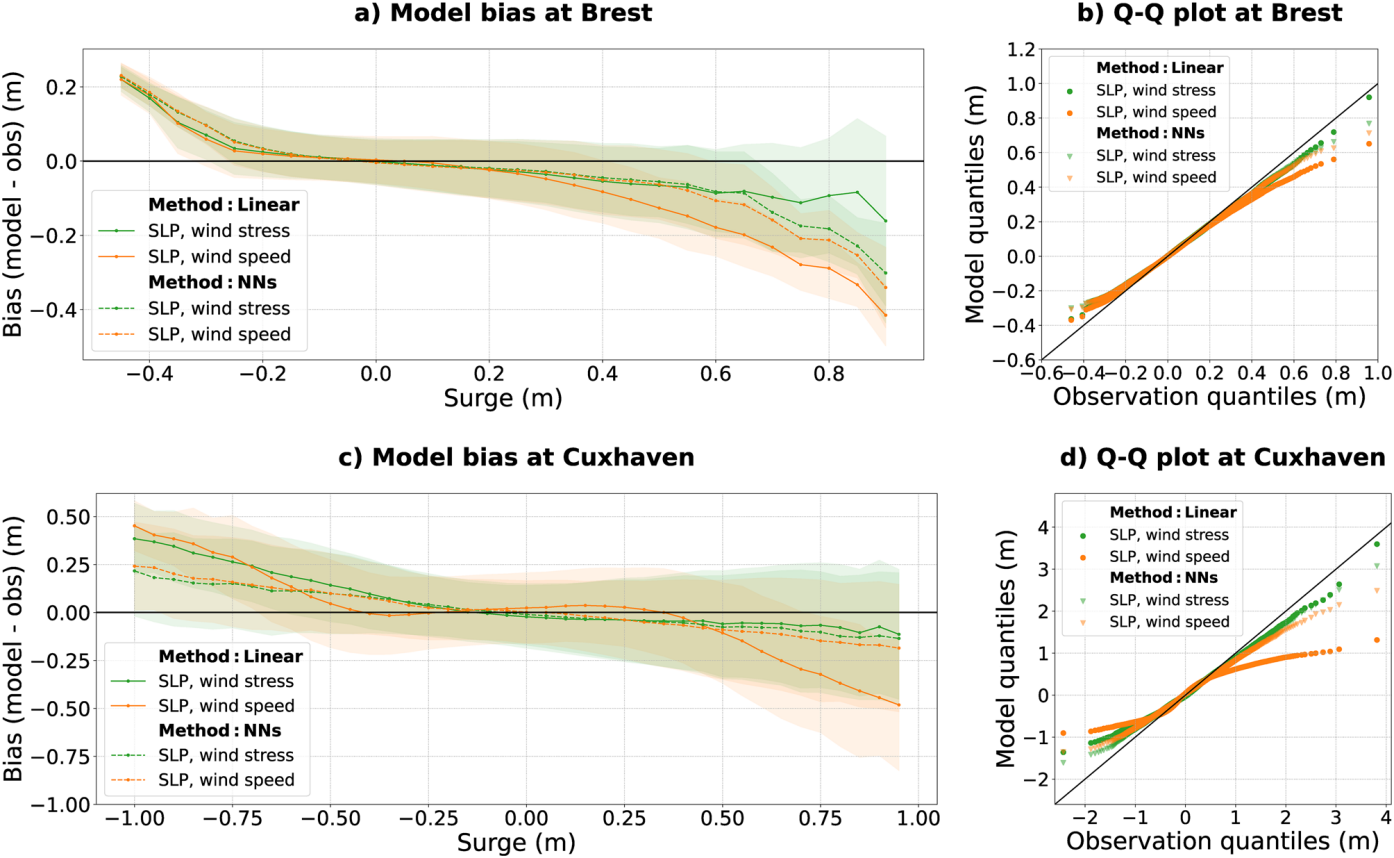
Bias for surges computed over the period 1994–2020 for (**a**) Brest and (**c**) Cuxhaven, using two different methods: multiple linear regression (full line) and Neural Networks (dashed line). Various local predictors were utilized: SLP and wind speed (orange), SLP and wind stress (green). The shaded areas correspond to the $\pm 1\sigma$ confidence interval. QQ plots are presented for (**b**) Brest and (**d**) Cuxhaven, using same methods and predictors: multiple linear regression (points) and neural networks (triangles).

**Figure 4:**
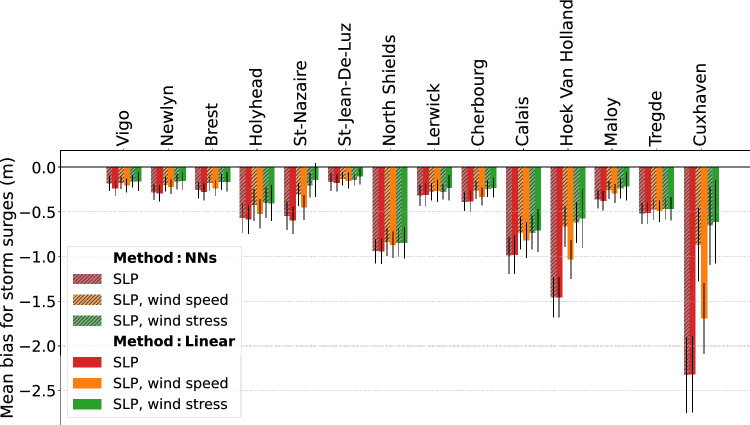
Mean bias for storm surges (i.e., larger than the 99.9th percentile of surges) computed over the period 1994–2020 at the 14 stations, for two different models: multiple linear regression (solid bars) and Neural Networks (hatched bars), and for various predictors: SLP alone (red), SLP combined with wind speed (orange) and SLP combined with wind stress (green). The error bars (black lines) represent the standard deviation.

To investigate how extreme surges are underestimated, the mean bias for storm surges (i.e., the bias between the modeled and observed extreme surges, defined as larger than the 99.9th percentile; for more details, see the Methods section) are computed over the period 1994–2020 at the 14 stations, and presented on Fig. 4. Regardless of the method (linear or NNs) and the predictors used (SLP, wind speed, wind stress), positive storm surges are always underestimated at all the stations (biases are always negative, as shown in Fig. 4). In the best configuration (see the green bars in Fig. 4, see also the Q–Q plots at Brest and Cuxhaven in Fig. 3), the bias varies from $-0.11$ to $-0.85$ m depending on the station. Note that these values should be interpreted according to the amplitude of the storm surges, which varies greatly depending on the station. For example, a bias of $-0.61$ m at Cuxhaven is relatively small, given the large storm surges at this station (the 99.9th percentile of surges reaches 2.08 m at Cuxhaven). The relative bias represents 24%, similar to the relative bias at Brest (also 24%, but for a smaller bias of $-0.17$ m).

### Wind stress versus wind speed

First, the mean bias for extreme surges is generally reduced when using the wind stress, rather than the wind speed, as a predictor in addition to the SLP (Figs. 2 and 4). This is particularly true for the linear regression model (the RMSE for storm surges decreased from 0.56 to 0.41 m on average at all the stations, and the bias decreased from $-0.56$ to $-0.36$ m). This result can be easily explained, as the relation between residual surge levels—once the classical inverted barometric effect is removed—and the wind stress is close to a linear relation, which is not the case between the residual surge levels and the wind speed. At Brest, during the Ulla storm, the modeled peak surge reaches 0.8 m with the wind stress as a predictor, against only 0.5 m with the wind speed as a predictor (Fig. 2a, 0.96 m for the observation). Similarly, at Cuxhaven during the Anna storm, the peak surge exceeds 3 m using wind stress, against 1.2 m using wind speed (Fig. 2b, 3.8 m for the observation). In both cases, the modeled peak surges are closer to the observations using the wind stress rather than the wind speed (Fig. 2). Overall, at all the stations, the mean bias for storm surges is reduced when using the wind stress rather than the wind speed (Fig. 4). The largest improvement occurs at Cuxhaven with a bias reduction from $-1.7$ to $-0.61$ m. Note that NNs models also perform better when using the wind stress, rather than the wind speed as a predictor at most of the stations (13 of 14, see Fig. 4). However, the improvement is often modest and not substantial.

Using the wind stress rather than the wind speed, NNs and linear models perform similarly (Figs. 2 and 4). This might appear to contradict the existing literature^14,17^ reporting improved NNs performances compared to linear regressions. However, the wind speed, and not the wind stress, was used as a predictor. Present results strongly suggest to more robustly consider the wind stress and not the wind speed as a predictor, when comparing a NNs (or any other model) with a linear model.

At most of the stations, this wind stress effect mostly contributes to the extreme surge, to enhance the peak surge, whereas the SLP contributes to the low-scale variability of the surges. For example, at Brest during the Ulla storm, the peak surge reaches only 0.4 m with the linear model using SLP alone but reaches more than 0.8 m with the linear model using SLP combined with wind stress (see the red and green curves in Fig. 2a). The contribution of the wind stress is even larger at Cuxhaven, where the SLP-based linear model is struggling to capture the low-scale variability of the surges (see the red and green curves in Fig.2b). Notably, in the North Sea, the shallower depths amplify the influence of the wind stress, as the water depth appears in the denominator of the Ekman stress contribution in the classical shallow water Saint-Venant equations. Overall, at all the stations, the wind stress mainly drives the peak surge, reducing the bias between the modeled and observed storm surges (see the red and green color bars in Fig. 4 to consider the effect of adding the wind stress to the SLP-based linear model). This result is fully consistent with analysis reported by Pineau-Guillou et al.^36^: storm surge events generally display both fast-time and slow-time components, with the wind stress contributing mostly to the fast-time component.

The remaining bias (ranging from $-0.1$ to $-0.85$ m depending on the stations, see Fig. 4) could stem from the relatively coarse resolution of the atmospheric reanalyses used in the present study (around 28 km and 1 h for ERA5, 75 km and 3 h for 20CRv3). Dangendorf et al.^2^ already pointed out that the low temporal and spatial resolution of the atmospheric forcing can contribute to the underestimation of the most extreme surges. While possibly less impacting large scale pressure fields, more localized and rapidly evolving strong winds are often underestimated in atmospheric models, and this underestimation can reach 7 m/s for winds of 30 m/s^37^. To address this issue, some studies apply a bias correction on the wind forcing data to enhance operational storm surge prediction^38^.

### Adding the waves

To investigate the role of waves in extremes, we computed the mean bias for storm surges, including or excluding the significant wave height as a predictor (Fig. S1 in the Supplementary). We found that the introduction of waves in the linear model reduces the bias on extremes at only 5 stations (Vigo, Cuxhaven, Newlyn, Tredge and St-Jean-De-Luz), and this reduction is modest (an average of 1.63 cm, which represents 1.9% of the 99.9th percentile at these stations). The significant role of the waves at these stations is probably due to the wave setup, i.e., an additional surge due to wave dissipation (mainly by wave breaking) in nearshore areas^39,40^.

However, at most of the stations (9 of 14), the introduction of the waves does not significantly reduce the bias on extreme surges modeling. Notably, NNs models show modest improvement, and the mean bias for storm surges slightly decreases from $-0.38$ to $-0.36$ m. This may be surprising, as it is well known that waves may play a significant role in most energetic events. For example, waves generated 10 cm of wave setup at La Rochelle (France) during the Xynthia storm^39^ and 40 cm at Arcachon Lagoon (France) during the Klaus storm^40^. Although we did not find a significant role of the waves in the models, this does not mean that the waves do not contribute to the surge for the two following reasons. First, all the variables are highly correlated, particularly the wind stress and the waves. For this reason, the wave effect may have already been absorbed when linking surges with wind stress (through an increased linear coefficient due to the wind-wave effect, compared to the wind-only effect). Second, the surge residual after removing the SLP and wind effects is still quite significant, reaching a few tens of cm (e.g., a standard deviation of 11 cm for residual storm surges at Brest, where storm surges are defined as those larger than the 99.9th percentile). This residual contains surges due to unaccounted drivers, such as waves, as well as other potential drivers such as temperature, oceanic circulation or river mouth discharge. The magnitude of the wave setup is quite small (from a few cm to several tens of cm in the most energetic events); thus, its contribution may be hidden in the residual signal, among other contributions of similar magnitude. Finally, the small role of waves is not contradictory to previous studies. For instance, Bruneau et al.^14^ considered waves as predictor, but did not quantify whether this additional predictor enhanced or not model performance.

### Extremes in historical atmospheric reanalysis

Since statistically reconstructed storm surges still provide robust estimates, we can now investigate how extremes are well represented (or not) in historical atmospheric reanalysis. Note that we are following the same approach as Dangendorf et al.^2^ did at Cuxhaven (North Sea).

**Figure 5:**
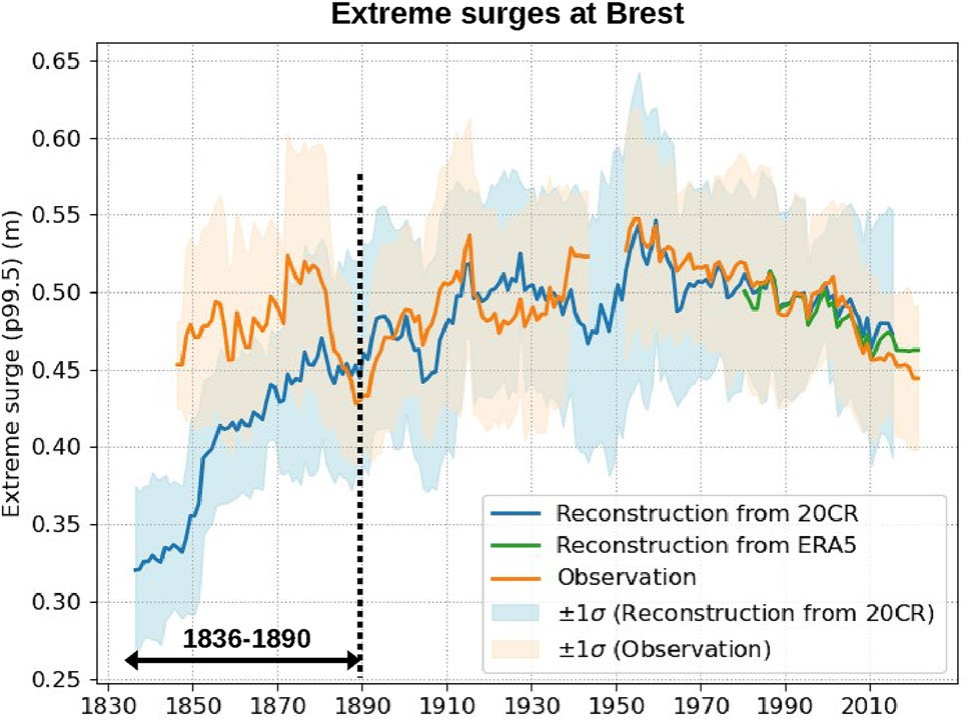
Extreme surges (99.5th percentile filtered on an 11-yr sliding window) at Brest, estimated from observations (orange) and modeled through a linear approach using predictors from 20CRv3 (blue) and ERA5 (green). Model were trained on data spanning the period 1990–2020. The shaded areas correspond to the $\pm 1\sigma$ confidence interval, associated to the 11-yr filtering process. Notable discrepancies between observations and reconstructions from 20CRv3 during the period 1836–1890 (black arrow) suggest an underestimation of extremes in 20CRv3 before 1890.

Here, we focus on the longest station with more than 150 years of data, i.e. Brest (France). The linear model is first trained over a recent period (1990–2020), with SLP and wind stress as predictors. The wind stress is computed following Eq. 5. Predictors are extracted locally from two atmospheric reanalyses: 20CRv3 and ERA5. We thus obtain two models with different coefficients for each reanalysis (20CRv3 and ERA5). We then reconstructed the storm surges over the reanalysis period, i.e., 1836–2014 for 20CRv3 and 1979–2021 for ERA5. Finally, we estimated the yearly extreme surges (defined as the 99.5th percentile of the annual surge time series) from the two reconstructions (20CR and ERA5) and observations. For the observations, annual percentiles were computed only when at least 75% of data were available yearly to avoid biases due to the seasonal cycle of surges. For the reconstructions, as storm surges are underestimated in models (see the remaining bias for storm surges in Fig. 4, ranging from 0.1 to 0.85 m depending on the station), we applied a classical bias correction^10,18,19^. The correction factor is computed as the mean ratio between the yearly observed and modeled extreme surges (99.5th percentiles) over a recent period (since 1980). Finally, the extremes are filtered on an 11-yr sliding window to eliminate the inter-annual variability. The results at Brest are presented in Fig. 5.

**Figure 6:**
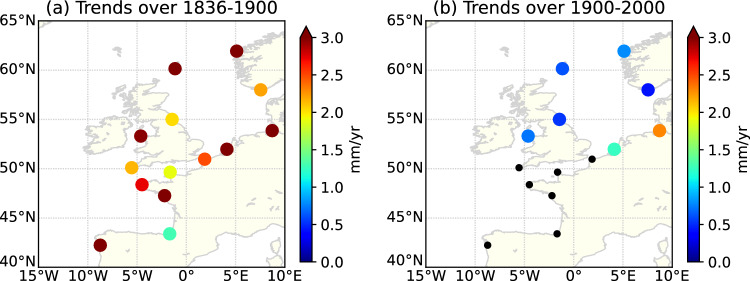
Trends in extreme surges reconstructed from 20CRv3 across the (**a**) 19th century (1836–1900) (**b**) 20th century (1900–2000). The positive trends over the 19th century may be attributed to a lower occurrence of extremes in the 20CR during that period. Map generated using Python Matplotlib 3.5.3 (https://matplotlib.org/).

Over the last century (since 1900), reconstructions based on both 20CRv3 and ERA5 agree well with the observations (Fig. 5). Reanalyses well capture the variability in extreme surges. Note that extreme surges reconstructed from 20CRv3 and ERA5 match despite the low temporal resolution of 20CRv3 (only 3 h for 20CRv3, against 1 h for ERA5).

In earlier periods (1836–1890), large discrepancies appear between the extreme surges reconstructed from 20CRv3 and observations (see the black arrow in Fig. 5). The strong positive trend in reconstructed storm surges from 20CRv3 (still during the period 1836–1890) is not in agreement with the observations at Brest, which do not display any significant trend (Fig. 5). This result at Brest is consistent with previous findings at Cuxhaven (North Sea), where Dangendorf et al.^2^ highlighted inconsistencies before the 1910s between extreme surges observed and those reconstructed from 20CRv2. Furthermore, we extended the reconstruction from 20CRv3 to all the stations and computed the trends over the 19th and 20th centuries. The positive trend over the period 1836–1890 is strong everywhere (Fig. 6a), with an average value of $3.4\pm 0.5$ mm/yr, whereas the trend is smaller over the period 1900–2000 (Fig. 6b), with an average value of only $0.5\pm 0.3$ 0.4 mm/yr, and half of the stations have no significant trend. The positive trend over the 19th century is probably due to a lower occurrence of extremes in 20CR during the 19th century, as already mentioned by Dangendorf et al.^2^. This could be due to fewer observations assimilated in the earlier periods.

As a result, for studies investigating past changes in intensity and frequency of storm surges, the 20CRv3 reanalysis may be fully appropriate over the 20th and 21st centuries, but not over the 19th century, due to a lower representation of extremes.

## Discussion

The wind stress is found to largely drives extreme surges, whereas the SLP contributes to the low-scale variability of surges (see Fig. 2 and the third result in the Results section).

**Figure 7:**
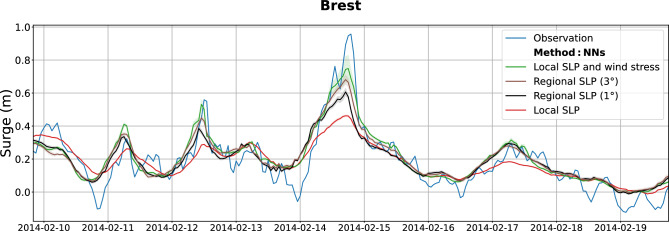
Reconstruction of the strongest storm surge event since 1994 at Brest (storm Ulla, 14 Feb. 2014) using Neural Networks and various predictors: local SLP (red), regional SLP at 1$^{\circ }$ (black) and 3$^{\circ }$ (brown), and local SLP combined with wind stress (green). Observations are depicted in blue.

Since local wind stress may be related to geostrophic winds associated to pressure gradients, we investigated whether NNs can be used to predict extreme surges without explicit wind information (wind and waves are no longer considered as predictors). To do so, we extracted the SLP locally (as in the Results section) and from larger areas around the tide gauges. Box sizes of 1$^{\circ }$ $\times$1$^{\circ }$, 3$^{\circ }$ $\times$3$^{\circ }$ and 6$^{\circ }$ $\times$6$^{\circ }$ were tested. Illustrated at Brest for a specific event (Fig. 7), NNs demonstrate improved performances as the SLP extraction area increases, up to 3$^{\circ }$ (no clear improvement is found with 6$^{\circ }$), to comparably perform compared to the NNs model using the local SLP combined with wind stress (see the brown and green curves in Fig. 7). This result can be extended to all the stations: the mean bias for storm surges is largely reduced when the SLP is extracted regionally at 3$^{\circ }$ (pink bars in Fig. S2 in the Supplementary, mean bias of $-0.41$ m), close to the performance of the linear model using local SLP and wind stress (green bars in Fig. 4, average bias of $-0.36$ m). Accordingly, the explained variance of the surges is similar when using NNs with the SLP extracted on a 3$^{\circ }$ box (Fig. S3 in the Supplementary) or using the linear model with the SLP and wind stress extracted locally (Fig. S3a).

Accordingly, NNs do not explicitly need local wind information to correctly model extreme surges when predictor is SLP extracted over a sufficiently large area. This suggests that the machine learning techniques are able to estimate the local wind stress effect from large-scale SLP data. Trained NNs thus statistically recover the properties of the internal atmospheric boundary layer, linking SLP gradients to the strength and orientation of the local wind stress. Wind stress is a key variable in air-sea interaction studies, and up to now, mainly estimated using bulk parameterizations based on the local surface wind data (possibly coupled with waves), with high uncertainties^37,41^. Here, NNs potentially embed an appropriate relationship between the wind stress and large-scale SLP data, indirectly discerned through its effect on sea level. Future investigations can be envisaged to delve into uncovering and interpret the inferred empirical NN relationships that locally link the wind stress with large-scale atmospheric pressure, to more precisely understand the dynamics of the internal atmospheric boundary layer and associated stratification during extreme events.

## Conclusion

We explored two statistical models for storm surge reconstruction in the North-East Atlantic: multiple linear regressions and NNs. Models were constructed using different predictors (SLP, wind speed or wind stress, and significant wave height). Various configurations are tested to investigate (1) which predictor among wind stress and wind speed best reduces the bias on extremes? (2) Does the addition of the waves as a predictor reduce the bias on extremes? (3) Do historical atmospheric reanalyses accurately represent extremes? We applied these models to 14 long-term tide gauges regularly distributed along North-East Atlantic coasts. The results are the following.

We found that extremes are always underestimated, regardless of the model, i.e, multiple linear regressions or NNs. There are several points to consider to reduce the bias on extremes.

Using the wind stress rather than the wind speed as a predictor significantly reduce the bias on extremes. This reduction is especially large when using a linear model. In such a configuration (wind stress as predictor) the linear and NNs methods perform similarly. A close linear relationship between the wind stress (and not the wind speed) and the surge residual is recovered, once the inverted barometric effect is removed. This result strongly suggests to systematically consider the wind stress, rather than the wind speed as a predictor, regardless the model.

The introduction of waves data as a predictor reduces the bias on extremes at 5 stations, although this reduction is modest. At most of the stations (9 among 14), the addition of waves does not significantly reduce the bias of predicted extremes, when solely using significant wave height as a first-order proxy for the wave setup.

Building on the reconstruction capabilities, we found that atmospheric reanalyses likely underestimate extremes over the 19th century. We found a strong positive trend in 20CRv3 reconstructed extreme surges over the period 1836–1890 which does not align with the observations at Brest. This suggests a lower occurrence of extremes during the 19th century in the 20CRv3. Consequently, using 20CRv3 data is not recommended for storm surge reconstruction in the North-East Atlantic during the 19th century, due to an underestimation of extremes. However, over the 20th and 21st centuries, 20CRv3 may be fully appropriate, as it effectively captures extreme events, including those occurring at the beginning of the century.

Finally, NNs can be applied without local wind information to correctly model extreme surges when the SLP is extracted on a sufficiently large area. NNs thus statistically recover the properties of the internal atmospheric boundary layer, linking SLP gradients to the strength and orientation of the local wind stress. Understanding these underlying relationships between the local wind stress and large-scale SLP data would be of great interest for air-sea interaction studies, which up to now, mainly link the wind stress to the wind speed (and not the atmospheric pressure).

## Methods

Two statistical methods (multiple linear regressions and Neural Networks) are used to model hourly storm surges (i.e., predictand) from metocean variables (i.e., predictors).

### Predictors

The predictors considered in this study are (1) the Sea Level Pressure (SLP), (2) the 10-m wind, (3) the wind stress and (4) the significant wave height. The wind speed and wind stress are both considered through their zonal and meridional components.

The wind stress is computed from the wind, using a classical bulk formula:

1 $$\begin{aligned} \tau _x= & {} \rho _{air} C_d \sqrt{u^2+v^2}u \end{aligned}$$

2 $$\begin{aligned} \tau _y= & {} \rho _{air} C_d \sqrt{u^2+v^2}v \end{aligned}$$

where *u* and *v* are respectively the zonal and meridional components of the wind, $\tau _x$ and $\tau _y$ are the wind stress components, $\rho _{air}$ is the air density and $C_d$ is the drag coefficient, expressed as a function of the wind speed^42^. This drag parameterization is used for the present study, but we ensured that the results were not sensitive to different $C_d$ parameterizations: a roughly constant $C_d$ value of $2.5 \times 10^{-3}$ led to the same results.

For each tide gauge, the predictors were extracted at different spatial scales: locally (at the grid point closest to the tide gauge) or regionally (on a 1$^{\circ }$ $\times$1$^{\circ }$, 3$^{\circ }$ $\times$3$^{\circ }$ or 6$^{\circ }$ $\times$6$^{\circ }$ box around the tide gauge). The spatial resolution of ERA5 is 0.25$^{\circ }$, which leads to 1 value for each predictor when extracted locally, and 16, 144 and 576 values respectively for each predictor when extracted on a 1$^{\circ }$, 3$^{\circ }$ or 6$^{\circ }$ box around the tide gauge.

The relationships between atmospheric predictors and surges are established simultaneously, without considering any temporal lag, as in recent papers^2,10,14,18,19^(that also do not consider any temporal lag). Indeed, we found that the correlation *r* between the surges and the predictors were almost the same (e.g. difference of $r^{2}$ smaller than 0.02 in average at all the 14 stations for SLP), whereas the predictors were lagged or not. Note that the lag was computed as the time shift, for which the correlation between the predictors and the surges is maximal.

In the present paper, ‘wind speed’ predictor refers to the *u* and *v* components and ‘wind stress’ predictor refers to the $\tau _x$ and $\tau _y$ components (see Eqs. 1 and 2).

### Multiple linear regression

We applied multiple linear regressions between metocean conditions (predictors) and hourly surge level observations (predictand) to predict hourly surge levels at a given tide gauge location. To investigate the role of the predictors in the surge, we used multiple linear regressions based on different predictors:

3 $$\begin{aligned} surge(t)= & {} a_1+SLP(t) \end{aligned}$$

4 $$\begin{aligned} surge(t)= & {} b_1+SLP(t)+b_2u(t)+b_3v(t)\end{aligned}$$

5 $$\begin{aligned} surge(t)= & {} c_1+SLP(t)+c_2\tau _x(t)+c_3\tau _y(t) \end{aligned}$$

6 $$\begin{aligned} surge(t)= & {} d_1+SLP(t)+d_2\tau _x(t)+d_3\tau _y(t) + d_4 h_s(t) \end{aligned}$$

where *SLP*(*t*) is the Sea Level Pressure, *u*(*t*) and *v*(*t*) are the wind components, $\tau _x(t)$ and $\tau _y(t)$ are the wind stress components and $h_s(t)$ is the significant wave height. The models were trained separately at each of the 14 tide gauges, to obtain unique coefficients (see section ’Model training and validation’).

When the predictors were extracted regionally (on a box around the tide gauge), rather than locally, we first performed a Principal Component Analysis (PCA) on each predictor, to best reduce the dimensionality and avoid spatial correlation effects between predictors. Indeed, extracting SLP from the 0.25$^{\circ }$ atmospheric model on a 3$^{\circ }$ $\times$3$^{\circ }$ box leads to 144 SLP time series that are highly spatially correlated as input for the model. We then used the first Principal Components (PCs) as predictors:

7 $$\begin{aligned} surge(t)= & {} e_0 + e_1 PC_1(t)+ e_2 PC_2(t)+...+e_nPC_n(t) \end{aligned}$$

where *n* is the number of first PCs that explain 95% of the variance. Note that the PCA decomposition is a classical approach when the predictors are extracted regionally^9–11,19^

### Artificial neural networks

We constructed a traditional Artificial Neural Networks (NNs) to predict hourly surges (predictand) from predictors. Artificial Neural Networks is the most common Neural Networks model and applies nonlinear relationships between predictors and predictand^15,43^. A 2-dimensional Convolutional Neural Network was also implemented without significant improvement, and was not further considered in this study. Other NNs, such as a Long Short-Term Memory layer (LSTM) or a Convolutional LSTM, have been implemented in other studies without exhibiting better performance than traditional Artificial Neural Networks. For example, Bruneau et al.^14^ reported no significant improvement in predictions using LSTM, although Tiggeloven et al.^15^ reported that LSTM generally outperforms other NNs at worldwide stations.

Here, we focused on the simplest and traditional NNs. Before training the model (see the section ’Model training and validation’), predictors were normalized between 0 and 1 by subtracting from each time series the minimum and then dividing the resulting time series by its maximum range. The NNs were implemented using the Python package Keras^44^.

The NNs architecture and hyperparameters were chosen similarly to those of Tiggeloven et al.^15^, who explored deep learning capabilities for surge predictions. The input layer contains as many nodes as predictors (for instance, 3 nodes when the predictors are the SLP and wind stress components extracted locally, and 144 nodes when the predictors are the SLP extracted on a 3$^{\circ }$X3$^{\circ }$ region). Then, the unique hidden layer contains 48 neurons and is activated using the ReLU activation function. After a dropout layer (with a dropout value of 0.2), the output layer is composed of one node that provides the surge prediction.

Note that we tested different NNs architectures and hyperparameters, to make our results more robust. Tests were conducted at all the stations, leading to similar results.

### Model training and validation

The total dataset (i.e., predictors and predictant time series) covers approximately 25 years, as the waves (one of the predictors) are available only over the 1994-2020 period. The dataset starts in 1994 and ends between 2015 and 2020, depending on the end of the sea level data record (see column 3 in Supplementary Table 1). In the present paper, the dataset period is simply referred to as 1994-2020.

The models are applied to the full dataset from 1994 to 2020, considering all the surges (not only the extremes), as the final objective is to extend data from tide gauges or numerical models^10,11,19^.

To verify that models did not overfit, each model at each location was validated via a cross-validation process. The predictors and predictand datasets were randomly split into a training dataset, which represented 80% of the total dataset (i.e., around 20 years), and a testing dataset, which represented 20% of the total dataset (i.e., around 5 years).

Models were constructed from the training dataset. For the linear model, we simply fitted a multiple linear regression between the predictand and predictors. For the NNs, the procedure is as follows. We first split the training dataset into a secondary training dataset, which represents 70% of the training dataset (around 14 years) and a validation dataset, which represents 30% of the training dataset (around 6 years). We chose a batch size of 12 weeks (2000 hourly values), i.e., the subset size of training data. An Adam solver is used to minimize the Mean Squared Error between the predicted and the observed surge values for 200 iterations maximum (called epochs). The Mean Squared Error of the secondary training dataset and the validation dataset (not used for training) are compared for each epoch, until they converge to a similar minimum value. Probabilistic predictions were generated using an ensemble of 20 NNs trained at each station and using random subsets, similar to previous studies^14,15^. Standard deviations of the ensemble (20 members) were also computed to estimate the confidence interval of the predictions.

Once the models were constructed, we checked that the performance of each model was similar using the testing dataset (unseen by the model) or the training dataset. This process was performed at least 3 times. Once the model was validated, the predicted surges were computed using the whole dataset.

### Statistical indicators

The following statistical indicators are used to evaluate the performance of each model:

- the bias, which refers to the differences between the model and the observations, considering all the surge values;
- the mean bias for storm surges, which refers to the bias between the model and observations, considering only extreme surges, i.e., those larger than the 99.9th percentile of the surges (computed over the entire time series),
- the Root Mean Square Error (RMSE) for storm surges, which refers to the RMSE between the model and observations, considering only extreme surges, i.e., those larger than the 99.9th percentile of the surges,
- the explained variance ($r^2$) between the model and the observations, where *r* is the Pearson correlation coefficient,
- the Quantile-Quantile (Q-Q) diagrams between the modeled and observed surges.

Note that the chosen value of the 99.9th percentile to select extremes is classically used^45,46^. Such a value allows us to select strong events that occur approximately 1.8 times per year. A sensitivity test was conducted on the 99th and 99.5th percentile, leading to similar results.

### Supplementary Information

Supplementary Information.

## Data Availability

The GESLA-3 sea level data set^20–22^ analyzed during the current study is available on the GESLA website. The ERA5 atmospheric hourly data^27,28^ are available on the Copernicus Climate Change Service (C3S) Climate Data Store. The 20CRv3 atmospheric data^29,30^ are available on the 20CR website. Support for the Twentieth Century Reanalysis Project version 3 dataset is provided by the U.S. Department of Energy, Office of Science Biological and Environmental Research (BER), by the National Oceanic and Atmospheric Administration Climate Program Office, and by the NOAA Physical Sciences Laboratory. The ResourceCode high-resolution wave hindcast database^31,32^ is available at Ifremer Sextant website. The NNs were implemented using the Python package Keras^44^.
