# Supplementary material for: Underestimation of extremes in sea level surge reconstruction
Source: Sci Rep. 2024 Jun 27;14:14875. doi: 10.1038/s41598-024-65718-6 (PMC11211459; doi:10.1038/s41598-024-65718-6)
Supplement: Supplementary file 1 — Supplementary Information. [file 41598_2024_65718_MOESM1_ESM.pdf]

Supplementary for “Underestimation of extremes in sea level surge reconstruction”

Ludovic Harter, Lucia Pineau-Guillou and Bertrand Chapron

Supplementary Table 1 – Main features of tide gauge records used in this study : station name, time span, number of years with data.

	Station name	Time span	Nb of years
1	Vigo	1943-2015	73
2	St-Jean de Luz	1942-2018	52
3	St-Nazaire	1821-2020	134
4	Brest	1846-2021	165
5	Newlyn	1915-2021	107
6	Holyhead	1964-2021	51
7	Cherbourg	1943-2020	50
8	Calais	1941-2021	56
9	Hoek van Holland	1900-2018	89
10	Cuxhaven	1917-2018	102
11	North Shields	1946-2021	68
12	Lerwick	1959-2021	63
13	Tregde	1927-2020	94
14	Maloy	1943-2020	76

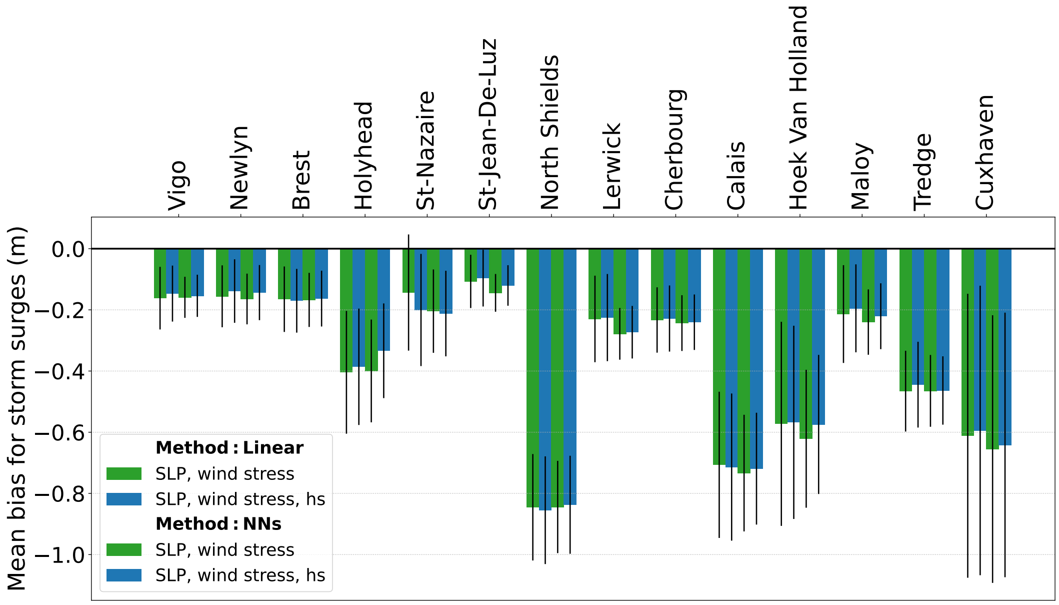

FIGURE S1 – Mean bias for storm surges (i.e., larger than the 99.9th percentile) computed over the period 1994-2020 at the 14 stations. Two different models are depicted : Multiple Linear Regression (solid bars) and NNs (hatched bars), and various predictors are used : SLP combined with wind stress (green) and SLP combined with wind stress and significant wave heights (blue). The error bars (black lines) indicate the standard deviation.

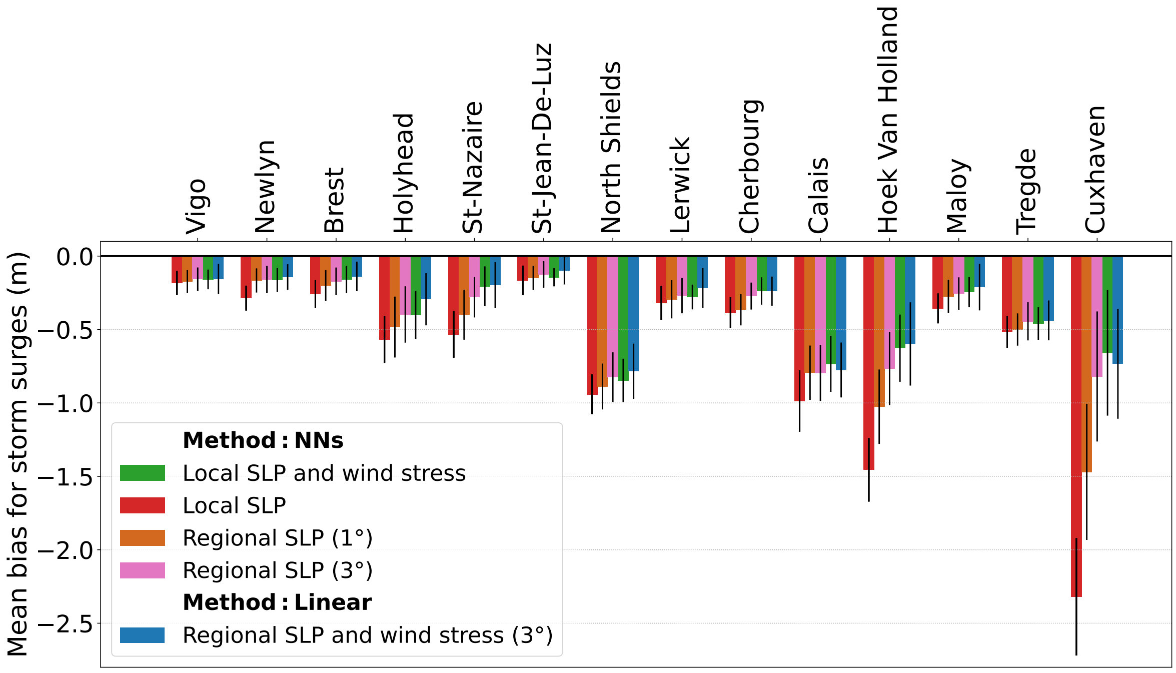

Figure S2 – Mean bias for storm surges (i.e., larger than the 99.9th percentile) computed over 1994-2020 at the 14 stations using both NNs (hatched bars) and linear models (solid bars). Predictors for NNs include local SLP (red), regional SLP at 1° (orange) and 3° (pink), as well as local SLP combined with wind stress (green). For the linear model, predictors are the 3° regional SLP and wind stress (blue). The error bars correspond to the standard deviation.

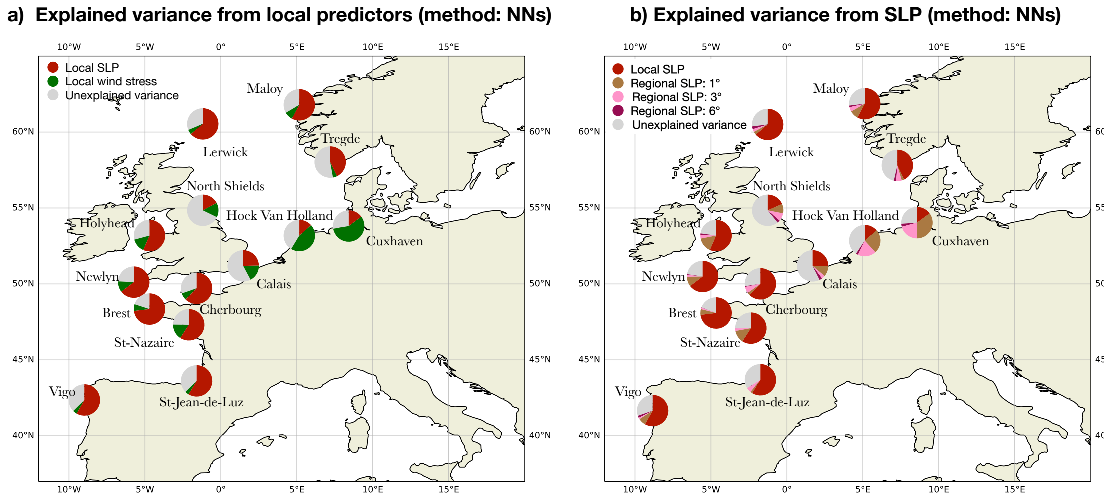

Figure S3 – Surges' explained variance from various predictors using NNs models : (a) locally extracted SLP and wind stress, (b) SLP alone extracted locally and regionally (within a 1°, 3° and 6° box around tide gauges). Map generated using Python Matplotlib 3.8.3 (https ://matplotlib.org/).
